# Primary desmoplastic small-round-cell tumor of the ovary

**DOI:** 10.1186/s43046-019-0001-4

**Published:** 2019-11-15

**Authors:** Ahmed Atef, Khaled Gaballa, Mohammad Zuhdy, Khalid Atallah, Wagdi Elkashef, Shadi Awny, Basma Gadelhak, Basel Refky

**Affiliations:** 1 Surgical Oncology Unit, Oncology Center, Mansoura University, 70 Gomhoriya Street, Mansoura, 35516 Egypt; 2 Pathology department, Faculty of medicine Mansoura University, Mansoura, Egypt; 3 Radiology department, Mansoura University hospitals, Mansoura, Egypt

**Keywords:** Ovarian cancer, Desmoplastic small-round-cell tumor, Case report, Neoplasm, Metastasis

## Abstract

**Background:**

Desmoplastic small-round-cell tumor (DSRCT) is an extremely rare and highly aggressive malignancy. It is of yet unclear origin, but it is assumed to be of a mesothelial origin based on its tendency for widespread metastasis in serosal linings.

**Case presentation:**

In this report, we describe a young female who presented with bilateral ovarian masses that mimicked the classic clinical picture of ovarian cancer. The patient had a cytoreductive surgery done in the form of total abdominal hysterectomy, bilateral salpingo-oophorectomy, omentectomy, pelvic peritonectomy, low para-aortic and bilateral iliac lymphadenectomy. Postoperative course was smooth with no adverse events. The final pathology report revealed desmoplastic small-round-cell tumor. Afterwards, the patient was referred to medical oncologist to receive her adjuvant therapy.

**Conclusions:**

DSRCT is still an unknown disease to us given the limited number of cases and poor survival. Given the lack of clear guidelines, treatment is offered based on the best available evidence and the collaborative effort of a multi-disciplinary team.

## Background

Desmoplastic small-round-cell tumor (DSRCT) is an extremely rare and highly aggressive malignancy that was first described in 1987 by Sesterhenn et al. [1]. Then, it was more elaborately described by Gerald and Rosai in 1989 [2]. It tends to have a predilection for adolescent males with an annual incidence of about 0.1 cases per million. It usually presents as intra-abdominal masses that might mimic gastrointestinal stromal tumors (GIST) or retroperitoneal sarcoma [3]. It is of yet unclear origin, but it is assumed to be of a mesothelial origin based on its tendency for widespread metastasis in serosal linings. An epithelial, neurogenic and blastomeric origin had also been reported [4]. This malignant entity with its atypical clinical, pathological, radiological features, aggressive course, and extensive intra-abdominal dissemination may confer a diagnostic dilemma when it is first encountered. Here, we describe a young female who was presented by bilateral ovarian masses that was later revealed to be DSRCT.

## Case presentation

A 22-year-old female presented with abdominal pain. She had no significant medical and surgical history and had only been married for 4 months. Abdominal ultrasonography showed bilateral ovarian masses. CT scan and diffusion MRI revealed bilateral malignant-looking ovarian masses with associated peritoneal deposits and retroperitoneal lymphadenopathy. Also, an anomaly of horseshoe kidney was noted. Tumor markers cancer antigen 125 (CA-125) and CA19–9, carcinoembryonic antigen (CEA), lactate dehydrogenase (LDH), alpha-fetoprotein (AFP), and beta subunit of human chorionic gonadotropin (B-HCG) were all within the normal range. Upper and lower gastrointestinal endoscopies were done and excluded the possibility of gastrointestinal primary. After informed consent, surgical exploration aiming at complete cytoreduction was performed. Intraoperative exploration showed both ovaries to be totally replaced by malignant masses, with ascites and pelvic peritoneal deposits. The diaphragmatic surfaces and para-colic gutters were free from disease. One of the ovaries was excised and frozen section examination was requested which suggested the possibility of dysgerminoma. Cytoreductive surgery was done in the form total abdominal hysterectomy, bilateral salpingo-oophorectomy, omentectomy, pelvic peritonectomy, low para-aortic and bilateral iliac lymphadenectomy (high para-aortic lymphadenectomy could not be done due to the presence of horseshoe kidney). Apart from the high para-aortic lymph nodes, no gross residue was left at all.

**Fig. 1:**
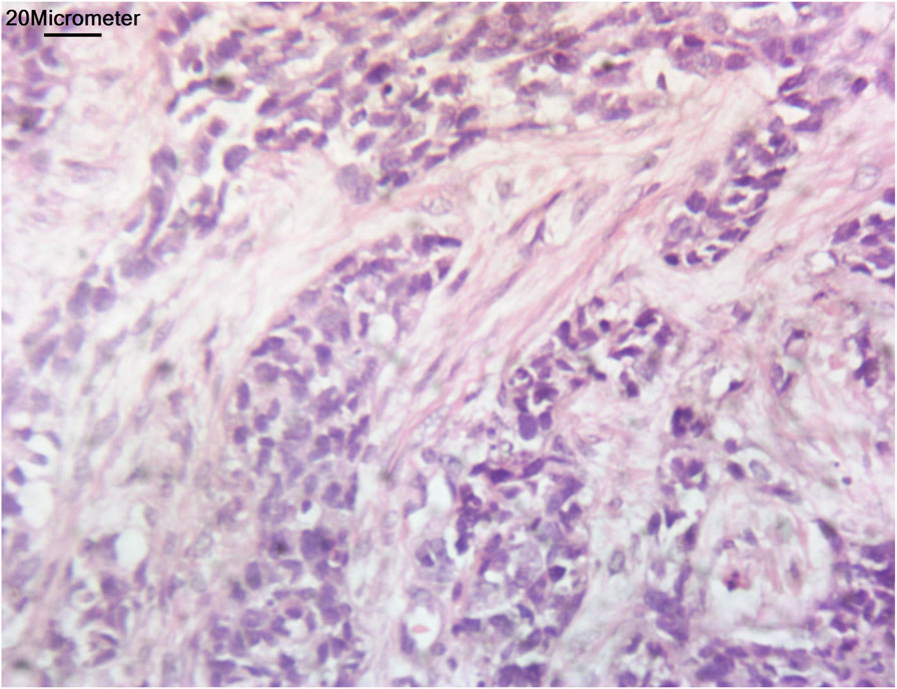
The tumor cells were small with marked degree of atypia and pleomorphism and high mitotic activity (Hx&E)

**Fig. 2:**
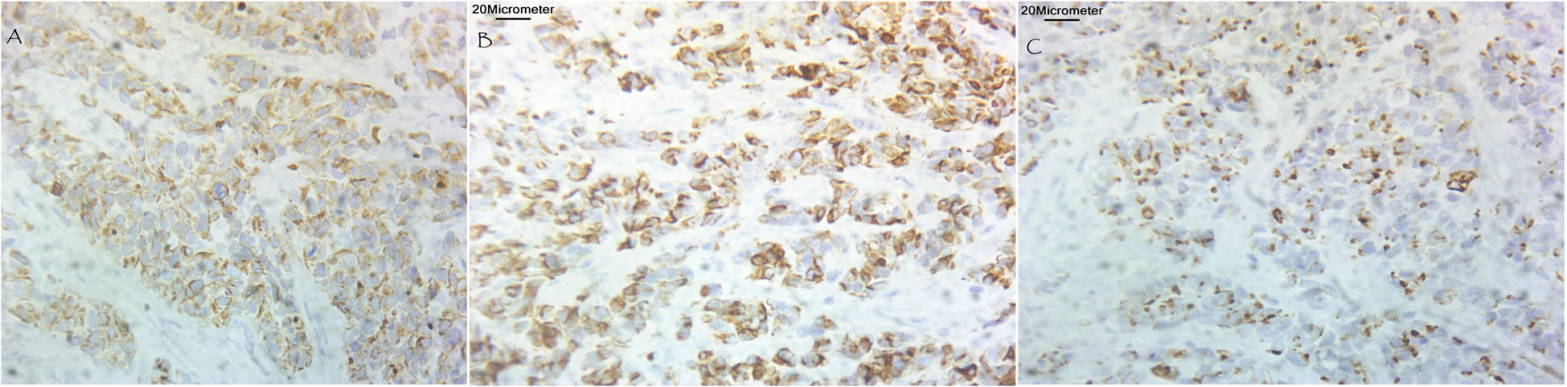
**a**–**c** Tumor tissue was positive for IHC desmin, cytokeratin, and WT-1

**Fig. 3:**
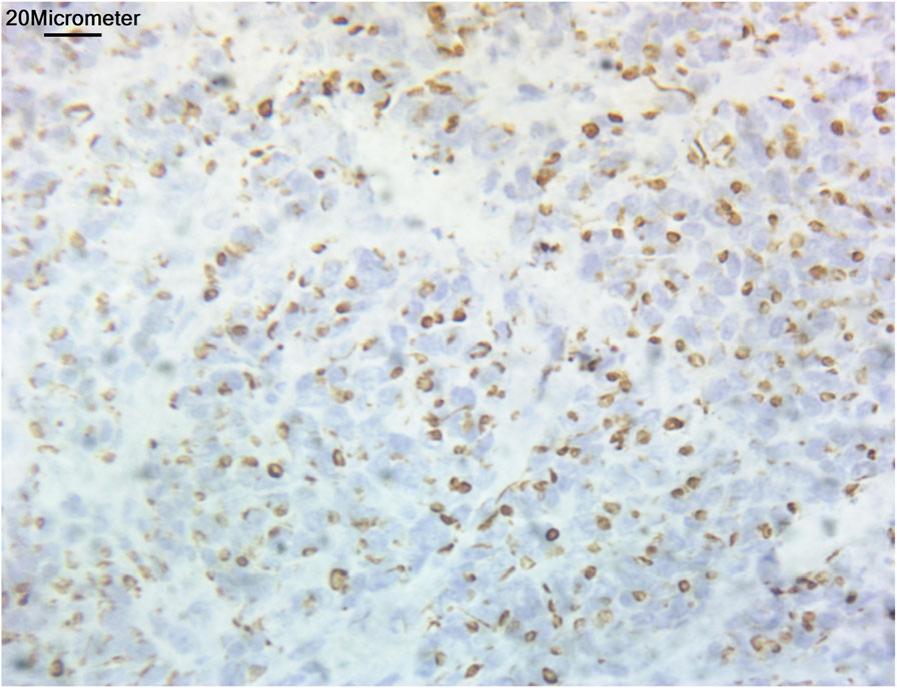
Tumor tissue was negative for IHC PLAP

Postoperative pathology examination revealed tumor tissue formed of malignant cells with intervening desmoplasia. The tumor cells were small with a marked degree of atypia and pleomorphism and high mitotic activity (Fig. 1). Three out of ten iliac lymph nodes and seven out of seven para-aortic lymph nodes were infiltrated by tumor tissue. Pelvic peritoneal nodules, uterine wall, and cervix were free from tumor infiltration. Immunohistochemical markers (IHC) were performed using monoclonal antibodies against pan-cytokeratin, cytokeratin 7, cytokeratin 20, placental alkaline phosphatase (PLAP), desmin, leukocyte common antigen (LCA), caudal type homeobox 2 (CDX-2), inhibin and GATA binding protein 3 (GATA-3), and Wilms’ tumor 1 (WT-1) (truncated human WT1 protein corresponding to N-terminal amino acids 1–181, FLEX, Monoclonal Mouse, Anti-Human Wilms’ Tumor 1 (WT1) Protein Clone 6F-H2 Ready-to-use (Dako Corporation)). Tumor tissue was positive for desmin and cytokeratin (Fig. 2) and negative for each of cytokeratin 7, cytokeratin 20, PLAP, WT-1, LCA, CDX-2, inhibin, and GATA-3 (Fig. 3) confirming the diagnosis of desmoplastic small-round-cell tumor.

The patient had an insignificant postoperative course. Afterwards, the patient was referred to a medical oncologist to receive the appropriate adjuvant treatment. Adjuvant chemotherapy (VACA-IE) protocol: vincristine (Oncovin), doxorubicin (Adriamycin), cyclophosphamide (Cytoxan), doxorubicin (Adriamycin), ifosfamide (Ifex), and etoposide (Vepesid) respectively were ensued soon after full recuperation. Patient is still receiving the proposed chemotherapy protocol, and she is planned to receive adjuvant external beam radiation therapy.

## Discussion

DSRCT is a rare and highly aggressive tumor that commonly affects the abdomen and or pelvis of young males. In their cases series, Al-Ibraheemi et al. reported 16 cases of extra-abdominal DSRCT. These reported sites included the head, neck, intracranial, thigh, axilla/shoulder, inguinal, paratesticular, intraosseous, and uterine body [5]. Moreover, Lae et al. reported DSRCTs originating from the ethmoidal sinus and soft tissue of the scalp [6]. To the best of our knowledge, DSRCT was reported to be originating from the ovary in 18 cases [7]. Interestingly, two of them presented during pregnancy and labor. Most of the reported cases presented with bilateral ovarian involvement with widespread peritoneal nodular infiltration [8, 9].

Its early presentation is usually non-specific, with symptoms varying between vague abdominal pain, distension, and altered bowel habits. Such a tendency to late presentation accounts for its difficult management, as it often manifests in advanced disease stage. Radiologic findings usually describe abdominal masses of variable sizes in association with peritoneal deposits, omental cakes, and ascites, findings that are usually found in the advanced colon, ovarian, and gastric cancer, hence the encountered diagnostic difficulty [10]. Differential diagnosis of small-round-cell tumor includes lymphoma, Ewing sarcoma, medulloblastoma, Wilms’ tumor, synovial sarcoma, neuroblastoma, and embryonal rhabdomyosarcoma [11]. Diagnosis is often confirmed after ultrasonographic- (U\S) or computerized tomography (CT)-guided biopsy from these lesions. Tumor markers’ elevation is non-specific. Only immunohistochemistry (IHC) and cytogenetic study are the tools that can confirm the diagnosis [12, 13]. The typical histopathological appearance of DSRCTs includes large necrotic area, sharply demarcated nests of different sizes containing small round cells with hyperchromatic nuclei and scanty faint eosinophilic cytoplasm, or spindle cells embedded in a desmoplastic stroma. These cells mostly exhibit epithelial, mesenchymal, and neural markers like cytokeratin, desmin, and smooth muscle actin (SMA) [14]. Desmoplastic small-round-cell tumor arises from mesenchymal cells of the abdominopelvic peritoneum. The gene fusion between Ewing sarcoma (*EWS)* and *WT1* genes resulting in the characteristic translocation t(11;22)(p13;q12), is the unique molecular hallmark and no other genetic factor has been linked to this aggressive tumor [15]. Horseshoe kidney is the most common fusion anomaly of the kidneys accounting for 0.25% of the population [16]. One of the major concerns that affected the treatment decision in the present case is the encountered technical difficulty of retroperitoneal lymph node dissection. Also, the radiation oncologist should have a good radiotherapy field planning to avoid radiation nephritis as most of the renal parenchyma overlies the para-aortic lymph nodes. In the abovementioned case, the patient was presented by bilateral ovarian masses, a finding that usually first directs the oncologist’s attention to the possibility of either Krukenberg tumor or primary ovarian cancer [17]. Indeed, this case was handled as such and it was only the final pathology that revealed the diagnosis of this rare tumor. In this case, a complete surgical staging—as if it was a case of primary ovarian cancer—was done given the presenting intraoperative situation and the result of frozen section analysis. Young age in and of itself might raise the suspicion of such malignancy, yet the rarity of the disease should keep us in the lane of common differential diagnoses for such age in an algorithmic fashion. It should be noted that until now there is still no consensus regarding the optimal management of this disease. The treating physician should follow the previously reported multimodality treatment to achieve the best disease response [7]. Based on the available data from the literature, a multimodality therapy of neoadjuvant multiagent chemotherapy followed by cytoreductive surgery and external beam radiotherapy is now considered the standard of care for those patients without extra-abdominal spread [18]. Honoré et al. reported improved overall and progression-free survival in patients treated with multimodality treatment including whole abdominopelvic radiotherapy (WAP RT) of 30 Gy. Fewer side effects were encountered with intensity modulated radiotherapy (IMRT) than two and three dimensional radiotherapy [19]. Despite relapse which was reported in most of the patients during long-term follow-up, prolonged progression-free survival was best reported after multimodality treatment [20]. Prognosis is still dismal with 5-year survival barely exceeding 30%.

## Conclusions

DSRCT is still an unknown disease to us given the limited number of cases and poor survival. Efforts are being maximized as much as possible to better find the optimal management. Given the lack of clear guidelines, treatment should be offered based on the best available evidence and the collaborative effort of a multi-disciplinary team.

## Data Availability

All data generated or analyzed during this study are included in this published article.

## Abbreviations

AFP: Alpha-fetoprotein
B-HCG: Beta subunit of human chorionic gonadotropin
CA 125: Cancer antigen 125
CA 19-9: Cancer antigen 19-9
CDX-2: Caudal type homeobox-2
CEA: Carcinoembryonic antigen
CT: Computed tomography
DSRCT: Desmoplastic small-round-cell tumor
EWS: Ewing sarcoma
GIST: Gastrointestinal stromal tumor
Gy: Gray
IHC: Immune histochemical stains
IMRT: Intensity modulated radiotherapy
LCA: Leukocyte common antigen
LDH: Lactate dehydrogenase
MRI: Magnetic resonance imaging
PLAP: Placental alkaline phosphatase
SMA: Smooth muscle actin
WAP-RT: Whole abdominopelvic radiotherapy
WT-1: Wilms’ tumor-1
